# Space-Confined Hydrogel Particle-Based Biosensor for Early Warning of Aflatoxin B1 via Rapid Label-Free Fluorescence Detection of the *afl*D Gene

**DOI:** 10.1021/acs.analchem.5c04105

**Published:** 2025-11-19

**Authors:** Kawtar Ettayri, Hailong Zhang, Wenwen Tian, Lingliang Long, Yu Chen, Mengyao Ma, Man Shing Wong, Kun Wang, Jing Qian

**Affiliations:** † School of Chemistry and Chemical Engineering, 12676 Jiangsu University, Zhenjiang 212013, P. R. China; ‡ Department of Chemistry and Institute of Advanced Materials, 26679 Hong Kong Baptist University, Kowloon Tong, Hong Kong, SAR China; § Analysis and Testing Center, 12579 Southeast University, Nanjing 211189, P. R. China

## Abstract

Aflatoxin B1 (AFB1), produced by the Aspergillus species, is one of the most potent carcinogens, posing significant health risks through contamination of food supplies. Monitoring an aflatoxin biosynthesis gene such as the *afl*D gene provides an early warning mechanism to prevent contamination. However, traditional methods for gene detection have limitations, including the need for sophisticated equipment and slow reaction kinetics, making them unsuitable for rapid and on-site testing. To address these challenges, we developed a novel biosensing platform based on space-confinement mechanisms using hydrogel particle arrays for the detection of the *afl*D gene. This system confines reactants within the outer water layer of poly­(ethylene glycol) carboxylate (PEG–COOH) hydrogel particles, significantly enhancing reaction kinetics by increasing local concentrations of the *afl*D gene. By incorporating graphene oxide to reduce background interference, the platform further improves detection specificity. The fluorescent probe used in this platform is a newly synthesized *V*-shaped dicationic fluorophore (VLM), which binds selectively to double-stranded DNA (dsDNA) and offers enhanced fluorescence upon binding to the *afl*D gene. This space-confinement-enhanced system detects the *afl*D gene with high sensitivity and rapid response, outperforming conventional methods by accelerating detection rates up to 240 times. The biosensor achieved a detection limit of 19.05 nM and a linear range of 50–1000 nM and required only 15 s for detection. This platform exhibited excellent sensitivity and specificity for the *afl*D gene, with high potential for real-time, on-site monitoring.

## Graphical abstract

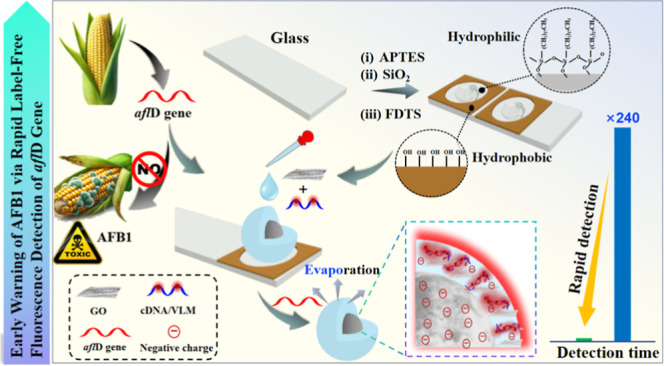

Aflatoxin B1 (AFB1), the most toxic member of the mycotoxin family, is well known for its hepatotoxic, carcinogenic, and mutagenic properties, even at trace levels.^1–4^ Recognized as a Group 1A carcinogen by the International Agency for Research on Cancer (IARC),^5,6^ AFB1 has emerged as a major global public health threat. Traditional detection methods for AFB1 predominantly rely on chromatographic and mass spectrometric techniques, such as high-performance liquid chromatography (HPLC) and liquid chromatography–mass spectrometry (LC–MS).^7–9^ In recent years, faster detection methods such as antibody-based immunosensors^10,11^ and aptamer-based aptasensors^12,13^ have emerged as promising alternatives. Despite their effectiveness in detecting AFB1 contamination, current methods predominantly operate after contamination has occurred. As a result, they are unable to prevent economic losses associated with the disposal of contaminated products and the disruption of supply chains. To address this limitation, researchers have begun shifting focus toward preventive detection strategies that target the molecular precursors of AFB1 production, particularly at the genetic level.^14,15^

The biosynthesis process of AFB1 is governed by a complex genetic pathway involving approximately 25–30 kinds of genes.^14^ These genes are clustered within a 70–75 kilobase (kb) region on chromosome III of the fungal genome, and they encode enzymes necessary for the biosynthesis of aflatoxins through a series of enzymatic reactions.^14–18^ Among these genes, the *afl*D gene plays a pivotal role in regulating the AFB1 biosynthesis process. Early detection of the *afl*D offers a valuable tool to prevent the production of AFB1 before it reaches dangerous levels of accumulation, which will significantly reduce the risks associated with AFB1 contamination in food and feed.^19^ Traditional quantitative methods for detecting the aflatoxin biosynthesis gene, such as the polymerase chain reaction (PCR)^20,21^ and reverse transcription PCR (qRT-PCR),^22,23^ often suffer from drawbacks such as long detection time, limited on-site applicability, and requirement of complex instrumentation. Currently, fluorescence biosensors have become increasingly attractive for *afl*D gene detection due to their operational simplicity, high sensitivity, and excellent selectivity. For instance, the Li group engineered a homogeneous Förster resonance energy transfer (FRET)-based biosensor for sensitive *afl*D gene detection by conjugating CdTe/CdS quantum dots (QDs) and gold nanoparticles (AuNPs) to amino- and thiol-modified hairpin DNA probes, respectively.^24^ Similarly, carboxyl-functionalized CdTe QDs^25^ and graphene quantum dots (GQDs)^26^ have been utilized as fluorescent labels for *afl*D gene detection, enabling the development of separation-free and magnetically controlled biosensors through conjugation with amino-modified single-stranded DNA (ssDNA). Although fluorescence biosensors offer significant advantages for the early warning monitoring of AFB1, their practical application faces fundamental challenges. Specifically, conventional approaches relying on functional-group modification of ssDNA probes incur high costs due to chemical labeling, while labor-intensive fluorescent labeling procedures significantly extend the fabrication time. Furthermore, the uncontrolled diffusion of target ssDNA in solution leads to inefficient molecular recognition and consequently prolonged detection time.^27^

To address these limitations, innovative biosensing platforms have been developed by mimicking natural space-confinement mechanisms.^28^ These systems confine recognition elements to cell-mimicking nanoscale domains, which not only enhance the molecular interaction efficiency through spatial control but also allow for the direct utilization of unmodified aptamers. This approach eliminates complex chemical modifications, simplifies the sensing process, and thereby reduces both cost and time.^29–31^ Hydrogels, as soft and tunable materials, provide an ideal microenvironment for housing abundant recognition elements in biosensor design. Most hydrogel materials are based on synthetic polymers such as polyethylene glycol (PEG), which can be cross-linked through covalent bonds and respond to a variety of physical and chemical stimuli including light, magnetic fields, and pH.^32,33^ Their porous 3D network facilitates analyte diffusion, while adjustable pore size and surface charge govern biomolecular interactions through outer water layer confinement, enabling internal diffusion or surface adsorption events.^34^ Recent advances highlight hydrogel platforms’ potential for biomolecule detection due to their biocompatibility and tunable porosity.^35^ However, conventional hydrogels suffer from slow analyte diffusion through their porous networks, which compromises sensitivity and prolongs response time.^35,36^ Consequently, nanostructured materials with engineered space confinement have emerged, significantly enhancing molecular recognition efficiency by concentrating analytes locally and restricting Brownian motion.^37–39^ Despite promising progress, integrating space-confinement effects into hydrogel biosensors remains underexplored, particularly for rapid, label-free detection of genes critical to AFB1 biosynthesis, which is essential for effective mycotoxin risk assessment.

In this work, we introduce a space-confined hydrogel microparticle array which enables early warning of AFB1 via rapid label-free fluorescence detection of the *afl*D gene (Scheme 1). This biosensing platform employed confinement of the analyte solution within the outer aqueous layer of polyethylene glycol carboxylate (PEG–COOH) hydrogel particles. This spatial confinement strategy effectively concentrated both the target *afl*D gene and the novel DNA-staining dye VLM, thereby accelerating the binding kinetics between the *afl*D-specific complementary DNA (cDNA) probe and the target gene compared to those of conventional bulk-phase methods. By restricting reactant diffusion, this approach optimized interaction efficiency. To enhance specificity, graphene oxide (GO) was integrated into the system because GO selectively adsorbed single-stranded cDNA probes while repelling double-stranded DNA (dsDNA) hybrids, significantly reducing background interference and ensuring high detection accuracy.^40^ These innovations enabled sensitive and rapid detection of the *afl*D gene, which is critical for proactive AFB1 risk management.

**Scheme 1:**
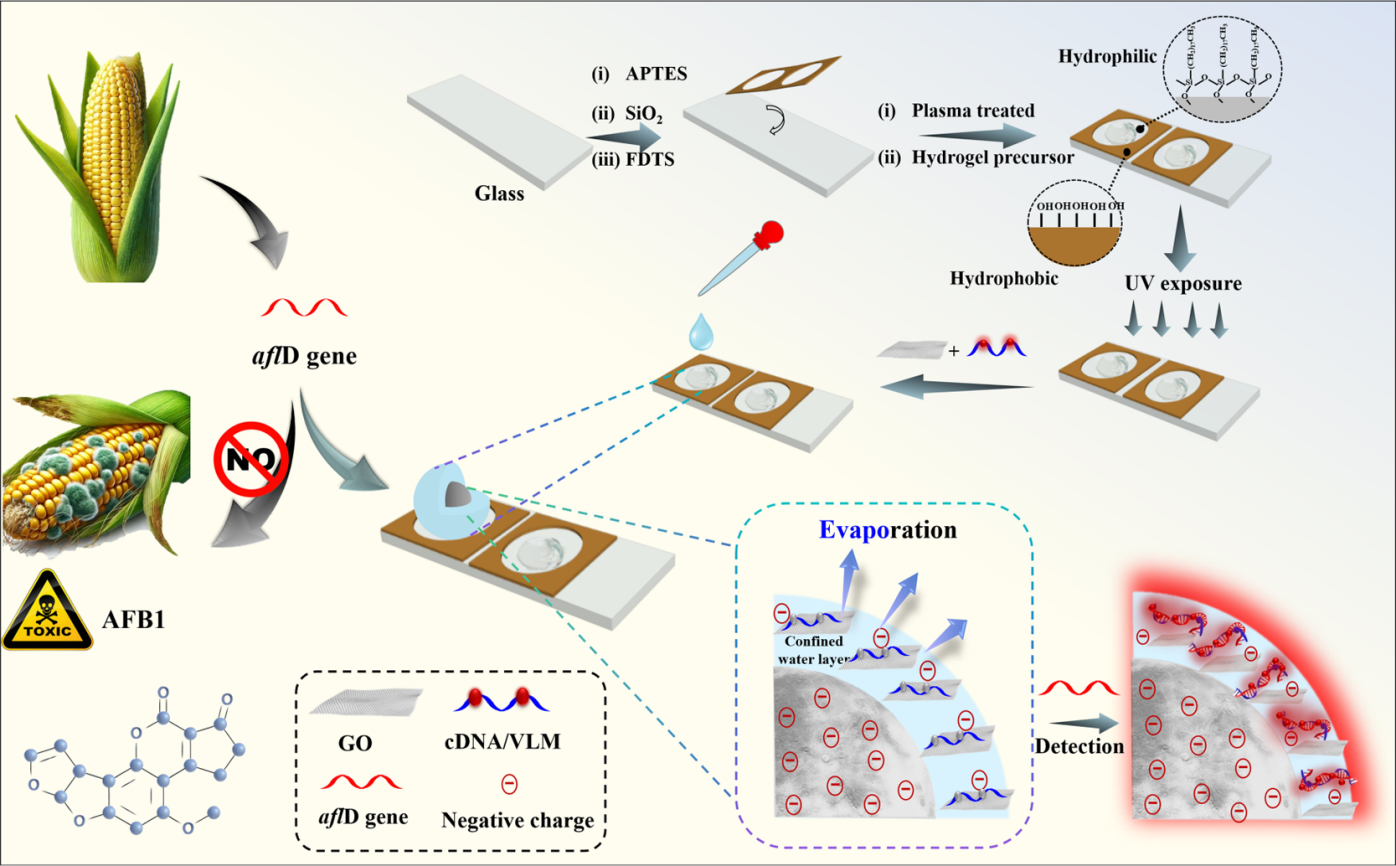
Schematic Illustrations of the Preparation Steps of the Hydrogel Particle Arrays and the Procedure for the Early Warning of AFB1 via Rapid Label-Free Fluorescence Detection of the *afl*D Gene

## Experimental Section

### Synthesis of GO

GO was produced according to Hummer’s process with some modifications.^41^ Initially, graphite powder (5 g) and NaNO_3_ (2.5 g) were mixed in 50 mL of concentrated H_2_SO_4_ (98.08 wt %) under an ice bath, followed by 30 min of stirring to achieve partial oxidation. The resulting mixture was thoroughly rinsed with deionized water and dried under vacuum at 50 °C. Then the pretreated graphite was added to a mixture of concentrated H_2_SO_4_/H_3_PO_4_ containing 9 g of KMnO_4_ and stirred continuously. Then the reaction mixture was cooled to room temperature. To stop the oxidation process, 0.675 mL of 30 wt % H_2_O_2_ was added. The product was then filtered and centrifuged at 5000 rpm for 30 min. It underwent multiple washes with water and ethanol, and the final residue was vacuum-dried to yield GO.

### Preparation of Superwettable Microchips

Acid-catalyzed silica sol (ACSS) was prepared following a reported method^42^ with slight adjustments. Five mL of tetraethyl orthosilicate (TEOS) was dissolved in ethanol (50 mL), followed by the introduction of 1.48 mL of a concentrated HCl solution (37 wt %) and 1.67 mL of H_2_O. The resulting mixture was sealed in a flask, stirred continuously for 4 h, and then allowed to age for 4 days at room temperature. Separately, 2 g of SiO_2_ was mixed into 80 mL of ethanol and stirred for 2 h to ensure proper dispersion. Afterward, 20 μL of the synthesized ACSS was introduced into the dispersion and stirred for an additional 1 h to yield the SiO_2_ suspension for further use. Glass substrates were thoroughly cleaned by sequential ultrasonication in acetone, ethanol, and deionized water and then dried using nitrogen gas.^43^ These substrates were treated with 2% ATPES, rinsed, and dried again. The glass substrates were repeatedly dip-coated 10 times with the SiO_2_ suspension. To create a superhydrophobic surface, the coated glass was immersed in a dilute 1H,1H,2H,2H-perfluorodecyltrichlorosilane (FDTS) solution (FDTS/toluene = 1:1000) for 30 min at room temperature, followed by rinsing with ethanol and heating for solidification at 80 °C for 20 min.^44^ The FDTS-modified superhydrophobic substrate was treated by plasma through a photomask using a 300 W power source for 15 min to create patterned superwettable microchips.

### Synthesis of the Hydrogel Particle Array

To fabricate the hydrogel particle array, a 5 μL precursor mixture was prepared, containing 20% (v/v) PEG diacrylate (PEG DA, MW 170.16), 40% (v/v) PEG (MW 200), 20% (v/v) PEG diacrylate (PEG DA, MW 170.16), 10% (v/v) acryl–PEG–COOH (23 mM, MW 3400) aqueous solution, and 10% (v/v) of a 2-hydroxy-2-methylpropiophenone solution in ethanol (655 mM) as the photoinitiator. This mixture was dispensed onto a superhydrophilic region and exposed to 365 nm UV light at an intensity of 60 mW/cm^2^ for 15 s to initiate polymerization. The resulting PEG–COOH hydrogel particles were stored in deionized water or maintained under high-humidity conditions until further use.^45^

### Fluorescence Detection of the *afl*D Gene Using the Hydrogel Particle Array

The fluorescent dye 4,4′-((1E,1′E)-(9-(2-(2-methoxyethoxy)­ethyl)-9H-carbazole-3,6-diyl)­bis­(ethene-2,1-diyl))­bis­(1-methylquinolin-1-ium) iodide (VLM), previously synthesized and characterized in our earlier study,^46^ was employed in this work. All of the oligonucleotides were diluted with Tris–HCl buffer (23 mM, pH 7.4). The dissolved DNA solutions listed in Table S1 were first heated for 5 min at 90 °C, followed by gradual cooling to ambient temperature to ensure proper hybridization before use. To construct the biosensing system, 3 μL of a mixture containing 100 μg·mL^–1^ GO and 10 μM cDNA/VLM complex was added to the hydrogel particles at room temperature and mixed for 20 min on the hydrogel particle-based biosensing platform. Then, 3 μL of different concentrations of the *afl*D gene were added to the platform, and the resulting fluorescence signal was immediately recorded to assess the biosensor’s response using laser confocal scanning microscopy (LSCM) with excitation at 543 nm.

## Results and Discussion

### Synthesis and Properties of VLM

The VLM molecule was synthesized as a *V*-shaped fluorophore featuring a bis-donor–acceptor architecture^46^ containing two quinolinium groups that function as potent electron acceptors connected by vinyl bridges to an electron-rich carbazole unit with exceptional emission properties.^47^ The VLM molecule gains two positive charges from these quinolinium moieties, which increases its affinity for the negatively charged phosphate backbone of DNA.^46,48^ This cationic property enhances the molecule’s solubility in aqueous settings, which is advantageous for biosensing applications, and fortifies its bond with DNA, causing the fluorescence intensity to significantly rise upon contact. By binding preferentially within the minor groove of dsDNA due to electrostatic forces, VLM achieves increased selectivity for dsDNA over ssDNA, regardless of the specific sequence.^46,49^ In comparison to other analogous probes, such as SYBR Green I that intercalates between DNA base pairs, VLM demonstrates several distinct advantages. Its excellent solubility in polar solvents, such as methanol, enables the preparation of stable and consistent stock solutions at 10.0 μM, ensuring high reproducibility in experimental assays. While VLM showed weak fluorescence in dilute aqueous solutions, a significant fluorescence enhancement was observed in the presence of dsDNA (Figure 1A) due to electrostatic interactions between its dicationic quinolinium groups and the negatively charged phosphate backbone. These interactions restricted intramolecular motions, thereby boosting fluorescence emission while suppressing nonradiative decay. Moreover, the *V*-shaped dicationic architecture of VLM promoted preferential binding within the minor groove of dsDNA rather than intercalation into the base pairs. This binding mode minimized perturbation of the DNA double helix, enhanced selectivity toward dsDNA over ssDNA, and ensured a stronger and more stable fluorescence response. Additionally, UV–vis absorption spectra in Tris–HCl buffer (23 mM, pH 7.4) displayed a broad band centered at ∼465 nm (Figure 1B), reflecting pronounced intramolecular charge transfer that contributes to the high signal intensity. Those features highlight the superior solubility, selective binding, and enhanced fluorescence performance of VLM, making it a promising fluorophore probe for sensitive and reproducible DNA detection.

**Figure 1:**
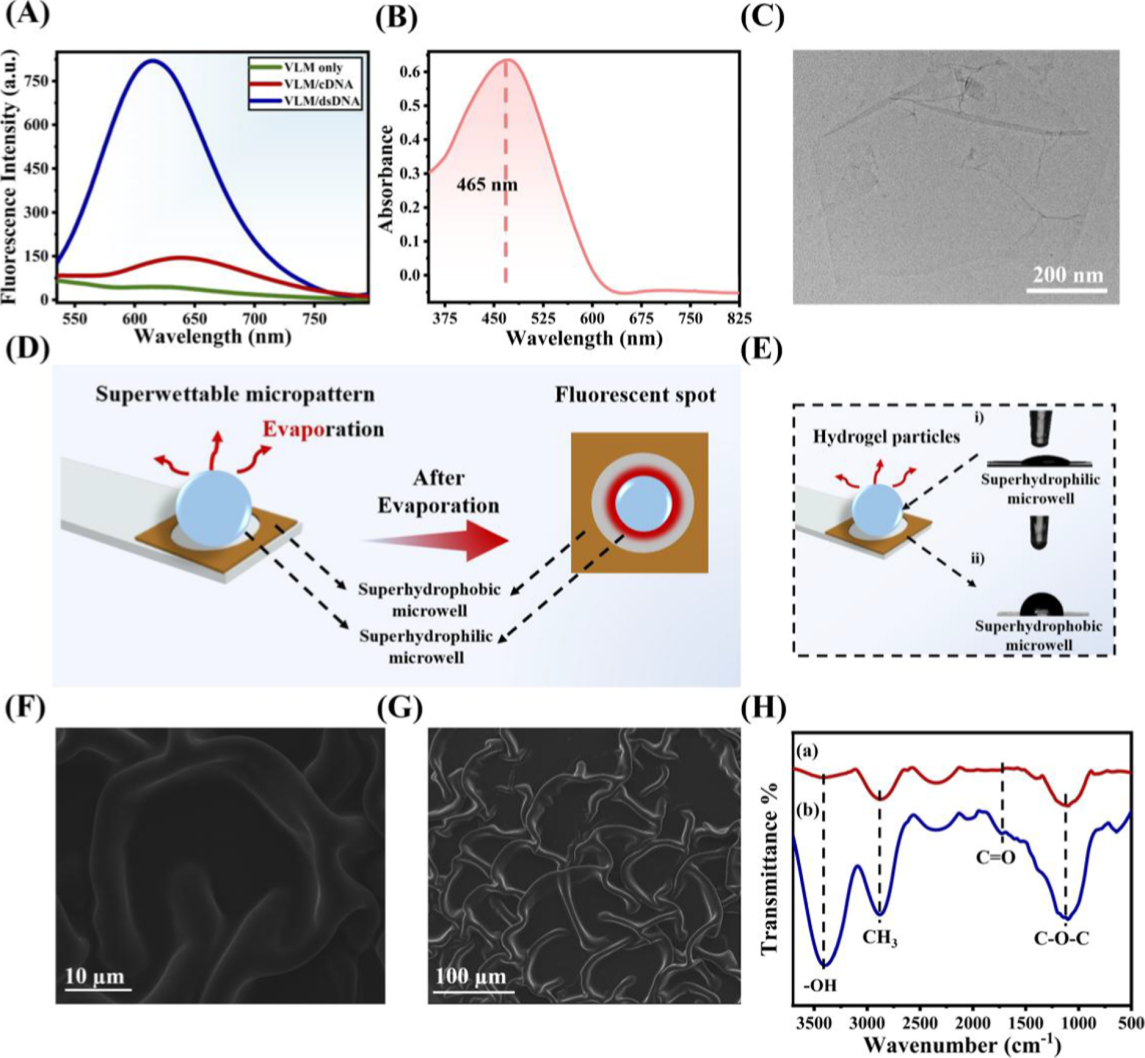
(A) Emission spectra of VLM (10.0 μM) in the absence and presence of 10 μM dsDNA and ssDNA measured in 23 mM Tris–HCl (pH 7.4). (B) UV–vis spectrum of VLM (10.0 μM). (C) TEM image of GO. (D) Schematic illustration of homogeneous spot deposition within superwettable micropatterns after droplet evaporation and (E) water contact angles of PEG–COOH hydrogel microparticles: (i) superhydrophilic and (ii) superhydrophobic substrate. SEM images of PEG–COOH hydrogel microparticles at (F) high and (G) low magnification. (H) FTIR spectra of (a) acryl-PEG–COOH precursor and (b) PEG–COOH hydrogel microparticles.

### Characterization of GO

GO nanosheets were synthesized following established protocols.^41^ The TEM image revealed their characteristic wrinkled and layered morphologies (Figure 1C), confirming successful exfoliation and preparation. These structural features are ideal for biosensing applications, as the high surface area and flexibility of GO nanosheets facilitate efficient ssDNA immobilization and signal transduction.

### Characterization of the PEG Hydrogel Particle Array

A patterned array of PEG hydrogel particles was fabricated by utilizing superhydrophilic microwells on a contrasting superhydrophobic surface. This difference in wettability enabled precise confinement of 3 μL of the hydrogel precursor solution within each microwell (Figure S1), allowing for the formation of a uniform PEG hydrogel array without cross-contamination after UV polymerization (Figure S2), as shown in Figure 1D. The hydrophilic nature of the hydrogel particles was confirmed through water contact angle measurements (Figure 1E),^50^ (i) The substrate demonstrated water repellency due to the modification of FDTS with a low surface energy (Figure 1E), (ii) Scanning electron microscopy (SEM) images of the dried PEG hydrogel revealed a compact and dense surface structure (Figure 1F), indicating the successful formation of the hydrogel matrix.^51^ Quantitative pore-size analysis was subsequently performed using the SEM images (Figure 1G) using ImageJ. After excluding very small artifacts (Area < 5 pixels), the hydrogel exhibited an interconnected porous network with an average pore diameter of 1.07 ± 0.76 μm (*n* = 231), a median diameter of 0.83 μm, and a size range of 0.32–5.84 μm. These pore dimensions were well suited for efficient analyte diffusion in our sensing application. Furthermore, poly­(ethylene glycol) diacrylate (MW 170.16) was employed to create this porous microstructure, and Fourier-transform infrared (FTIR) spectroscopy confirmed the presence of characteristic peaks corresponding to acryl–PEG–COOH (Figure 1H).

### Distribution of the *afl*D Gene on PEG–COOH Hydrogel Particles

To investigate how surface charge affected the spatial localization of the *afl*D gene, PEG–COOH hydrogel particles with negatively charged surfaces were synthesized (Scheme S1). The spatial distribution of the *afl*D gene on the hydrogel particles was examined using LSCM images. For the negatively charged PEG–COOH hydrogel particles, the cDNA/VLM complex was predominantly localized in the outer aqueous shell of the hydrogels. As this outer layer gradually evaporated, the concentration of cDNA/VLM in that region increased, leading to a corresponding increase in fluorescence intensity over time (Figure 2A,B). LSCM tomography images further confirmed that the cDNA/VLM complex remained predominantly localized at the outer regions of the hydrogel matrix, likely as a result of electrostatic repulsion between the negatively charged hydrogel network and the anionic cDNA/VLM complex (Figure 2C).

**Figure 2:**
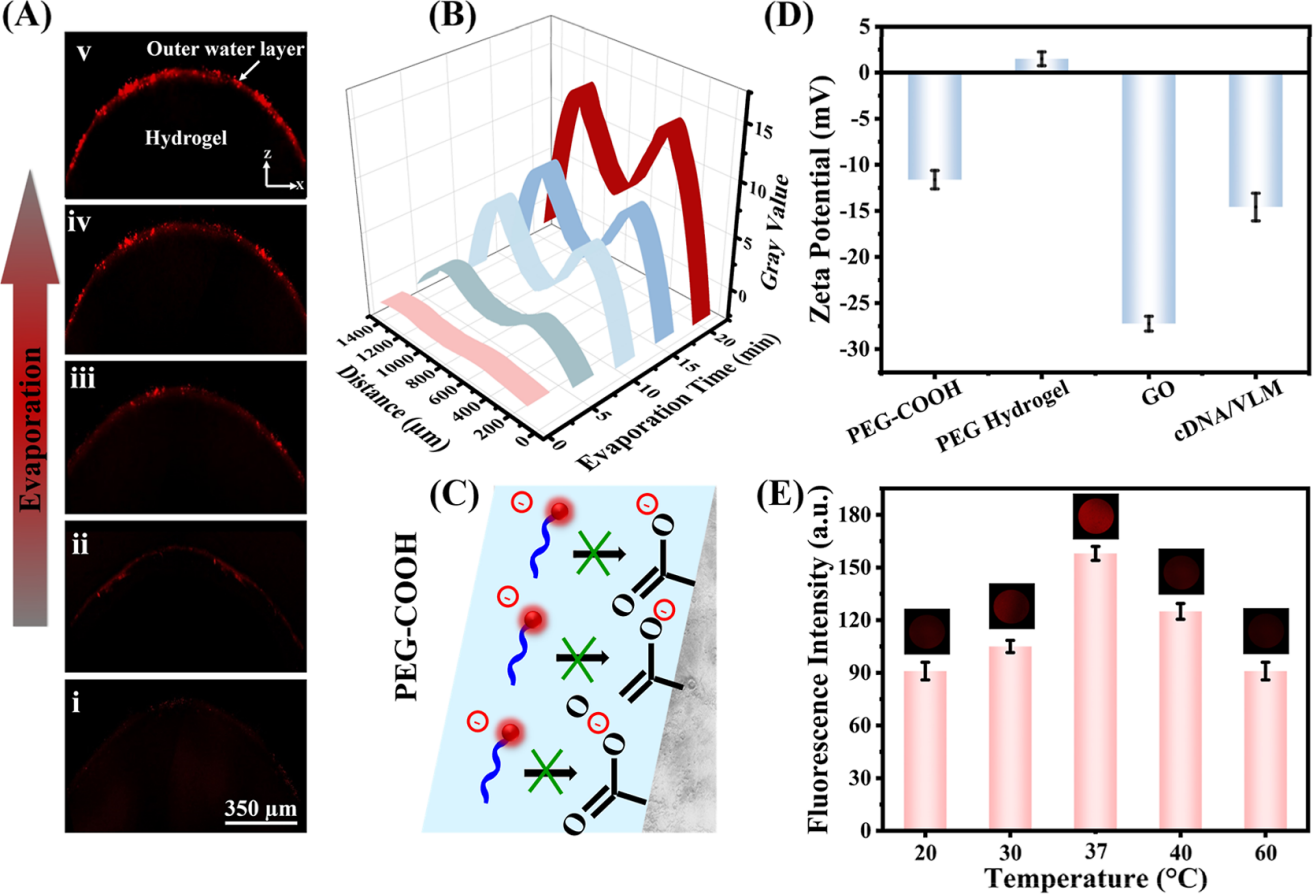
(A) Distribution of the cDNA/VLM complex on hydrogel particles during the evaporation process (from i to v) and the (B) corresponding cross-section analysis of cDNA/VLM droplets on hydrogel particles. (C) Schematic diagram of the interaction between hydrogel particles and cDNA/VLM complex. (D) Zeta potential of the PEG–COOH, PEG hydrogel, GO, and cDNA/VLM. (E) Effect on temperature for incubation between cDNA and VLM.

The zeta potential values of PEG–COOH hydrogel particles, PEG hydrogel particles, GO, and cDNA/VLM were also measured. The results showed that PEG–COOH, GO, and cDNA/VLM were all negatively charged, while the PEG hydrogel was nearly neutral (Figure 2D). The negative charges on both PEG–COOH and cDNA/VLM implied the presence of an electrostatic repulsive force between them. Additionally, incubation temperature optimization showed that the cDNA/VLM complex achieved the highest fluorescence intensity at 37 °C (Figure 2E). The cDNA/VLM complex was electrostatically enriched onto PEG–COOH hydrogel particles from the dilute solution, enabling fluorescent signal amplification. These findings demonstrated that negatively charged hydrogel particles effectively localize the *afl*D gene within the outer aqueous layer, thereby enhancing the biorecognition efficiency for biosensing applications.

### Design Principle of the Hydrogel Particle-Based Biosensor for the *afl*D Gene

GO effectively suppressed the fluorescence of the cDNA/VLM complex through a fluorescence resonance energy transfer (FRET) mechanism.^52^ Upon the introduction of the target *afl*D gene, the fluorescence signal was restored due to the formation of the cDNA/VLM/*afl*D gene complex.^53^ The compact structure of the PEG–COOH hydrogel surface prevented cDNA/VLM from entering the interior of the hydrogel. However, the fluorescent bright shells due to the space-confined effect of cDNA/VLM could be observed on the negatively charged PEG–COOH hydrogel surface, attributable to the spatial confinement of the cDNA/VLM complex. This effect was mainly caused by the electrostatic repulsion between the negatively charged hydrogel and the cDNA/VLM/*afl*D complex, which inhibited its diffusion into the inner hydrogel layers. These results underscore the critical role of surface charge in achieving effective molecular confinement. Additionally, the size of the hydrogel pores played a significant role in the confinement efficiency. The optimal confinement was achieved through a synergistic effect of both the charge and pore size. Additionally, the duration of the evaporation step played a key role in enhancing the enrichment efficiency. An evaporation time of 20 min was identified as optimal for maximizing both the confinement effect and detection sensitivity. Based on these findings, PEG–COOH hydrogels with smaller pores and a negative surface charge provided superior confinement, enriching the *afl*D gene on the surface of the hydrogel particles. Hence, PEG–COOH hydrogel particles were used to fabricate a hydrogel particle-based biosensor for *afl*D gene detection.

### Optimization of the Hydrogel Particle-Based Biosensor for the *afl*D Gene

GO was consistently localized on the surface region of the hydrogel particles for up to 45 min (Figure S3). To enhance the biosensor performance, both the GO concentration and the quenching of cDNA/VLM were optimized. As the GO concentration increased, the fluorescence intensity of cDNA/VLM on the hydrogel surface progressively declined, eventually stabilizing at a concentration of 100 μg·mL^–1^ GO, as shown in Figure 3A. The PEG–COOH hydrogel particles served as the confined reaction environment in this setup. Consequently, 100 μg·mL^–1^ GO was selected for the following experiments. Figure 3B shows that the fluorescence intensity of cDNA/VLM gradually decreased due to the quenching effect of GO, reaching a plateau at 20 min. Therefore, a GO concentration of 100 μg·mL^–1^ and a quenching time of 20 min were identified as optimal for the detection of the *afl*D gene.

**Figure 3:**
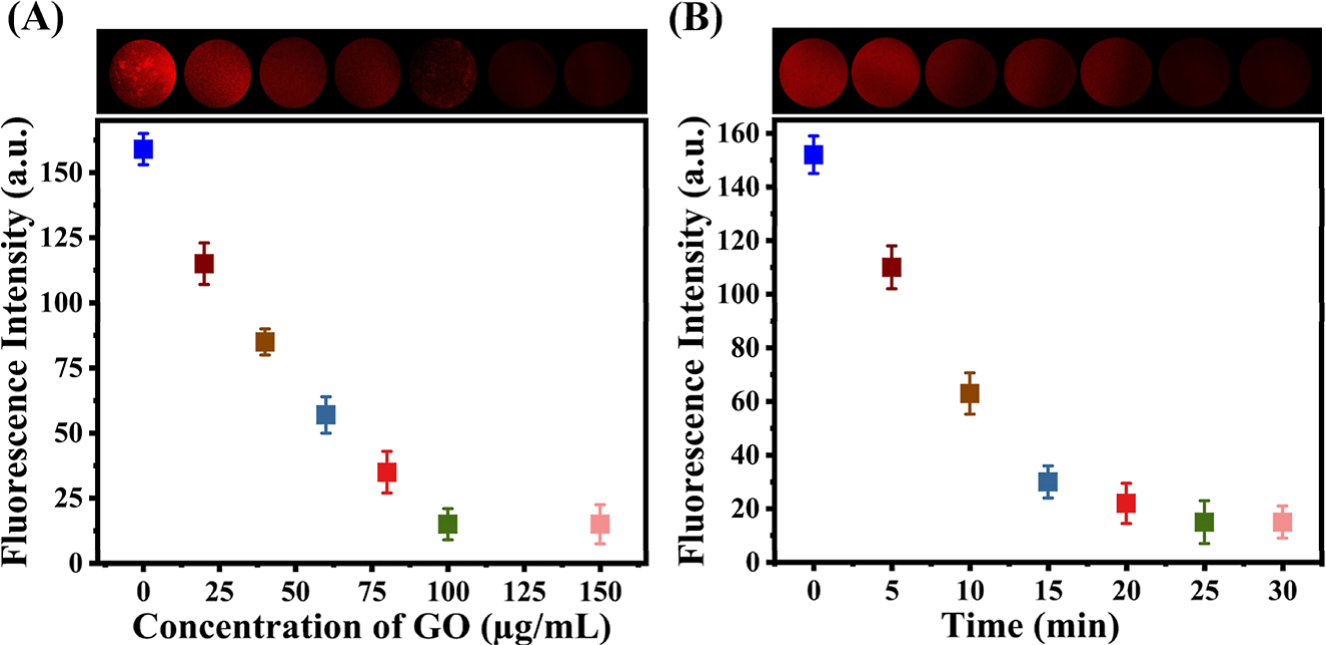
Effect of (A) GO concentration and (B) reaction time on the fluorescence responses of cDNA/VLM.

### Performance of the Hydrogel Particle-Based Biosensor for the *afl*D Gene

The performance of the fabricated biosensor based on the PEG–COOH hydrogel was thoroughly evaluated. As depicted in Figure 4A, fluorescence images revealed a gradual increase in intensity with the *afl*D gene concentrations ranging from 0.05 to 2000 nM. This enhancement in fluorescence intensity signified the formation of the cDNA/VLM/*afl*D gene complex. Furthermore, the fluorescence intensities remained consistent across three parallel experiments, demonstrating excellent analytical reproducibility. After analyzing the fluorescence intensity and the concentrations of the *afl*D gene (Figure 4B), a good linear relationship was obtained between the fluorescence intensity (*I*) and *afl*D gene concentration (*c*_aflD_), expressed as *I* = 39.68 + 0.11*C*_aflD/nM_ (Figure 4C). The biosensor exhibited a limit of detection (LOD) of 19.05 nM based on *S*/*N* = 3, with a linear detection range of 50–1000 nM and a high correlation coefficient (*R* ^2^) of 0.998. In this hydrogel particle-based space-confinement biosensing platform, the kinetics of DNA hybridization between cDNA and the *afl*D gene were remarkably fast, with the fluorescence recovery occurring within 15 s after adding the *afl*D gene at a concentration of 10 μM (Figure 4D). *afl*D gene detection in bulk solutions demonstrated significantly slower hybridization kinetics, with the maximum signal obtained only after 3600 s (Figure 5A). In contrast, the hydrogel particle-based biosensor represented a 240-fold acceleration compared with the conventional bulk aqueous system (Figure 5B). The substantial enhancement in DNA hybridization speed within the hydrogel particle underscored its ability to accelerate macromolecular interchain reactions, which involved diffusion in finite three-dimensional domains.

**Figure 4:**
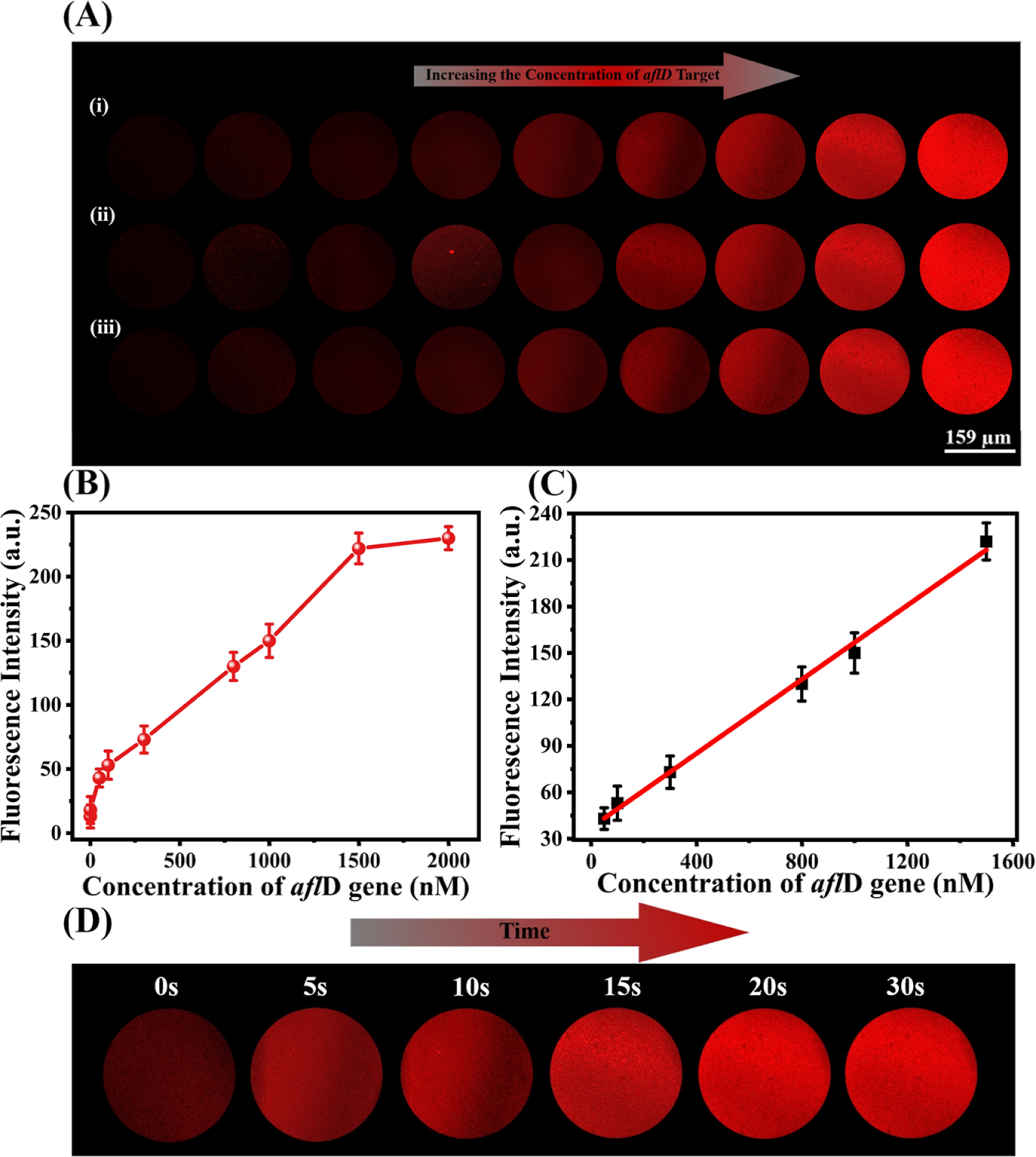
(A) The fluorescence images of *afl*D gene detection in three parallel experiments (i, ii, iii) using various concentrations (0.25, 50, 100, 300, 800, 1000, 1500, and 2000 nM). (B) The relationship between fluorescence intensity and *afl*D gene concentrations based on the VLM-based hydrogel particles (*n* = 3). (C) Linear curve of the fluorescence intensity versus the *afl*D gene concentration. (D) The real-time fluorescence images of the space-confinement biosensing platform after adding 1000 nM *afl*D gene.

**Figure 5:**
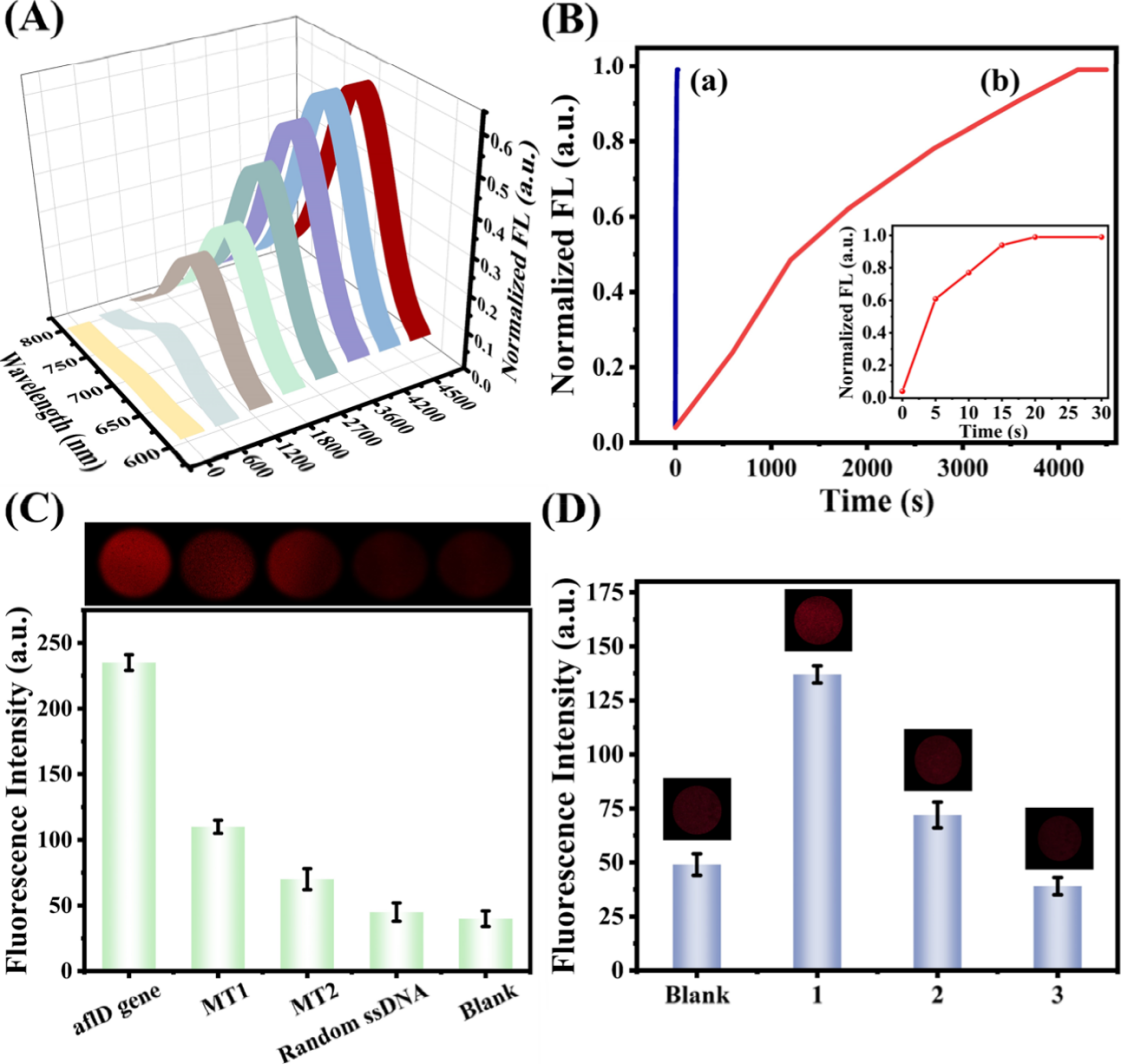
(A) Fluorescence intensity of the GO-cDNA/VLM/*afl*D gene complex system over time with 1000 nM *afl*D gene in bulk solution. (B) Real-time fluorescence response of 1000 nM *afl*D gene on (a) hydrogel particle-based biosensor and (b) in bulk. Inset: a zoomed view of the proposed biosensor. (C) Selectivity test of the hydrogel particle-based biosensor with the *afl*D gene, MT1, MT2, random-ssDNA sequence, and the blank (1000 nM for all DNA sequences). (D) Detection of the *afl*D gene in corn flour at the concentrations of (1) 10 μM, (2) 5 μM, and (3) 0.05 μM.

The DNA hybridization reaction is a fundamental process in many biochemical and biosensing applications. It involves an interchain reaction between complementary ssDNA that leads to the formation of dsDNA (eq 1). This reaction can be modeled as an association reaction governed by diffusion within a finite three-dimensional domain. The hybridization process can be represented as follows^54–56^

1 A+B→P

In this reaction, *A* and *B* are complementary ssDNA strands that combine to form the duplex DNA (*P*). The association is characterized by the rate constants *K*_on_ (association rate constant) and *K*_off_ (dissociation rate constant), representing the rates of duplex DNA formation and dissociation, respectively (eq 2). The overall rate of the reaction is governed by the balance between these two processes and can be expressed by the following rate equation

2 $$\frac{d\left[P\right]}{dt}=K_{on}\left[A\right]\left[B\right]-K_{off}\left[P\right]-K_{r}\left[P\right]$$

DNA hybridization proceeds through two primary kinetic-limiting phases: nucleation and diffusion. During nucleation, a short duplex forms, typically involving two to four base pairs that are hydrogen-bonded and stacked (Strand *A* × Strand *B*). This is followed by a rapid “zipping” phase, during which base stacking and hydrogen bonding are completed.^57^ This can simplify the rate equation, as shown in eq 3.

3 $$r=K_{on}\left[A\right]\left[B\right]$$

Applying the diffusion model and Fick’s first law, if the distance between Strand *A* and Strand *B* is *L*, the flux of Strand *B* is eq 4

4 $$J=-D_{B}\frac{\partial N_{B}}{\partial L}$$

In eq 5, *D*_B_ represents the diffusion coefficient, while *N*_A_ and *N*_B_ stand for the bulk concentrations of Strand *A* and Strand *B*, respectively, and *L*_A.B_ refers to the distance between Strand *A* and Strand *B* in the hydrogel particle-based biosensor (eq 5).

5 $$r=\frac{4\pi }{L_{A.B}}\left(D_{A}+D_{B}\right)N_{A}N_{B}$$

In the hydrogel particle-based biosensor for the *afl*D gene, the *afl*D gene was concentrated in the outer water layer of hydrogel particles. This led to an increase in the bulk concentration of *B* (*N*_B_) due to evaporation effects, thereby enhancing DNA hybridization kinetics. Moreover, the confined environment within the hydrogel induced a conformational change in the ssDNA, transforming it from a flexible, random coil into a stretched structure. The cDNA/VLM complex was enriched within this progressively concentrated and spatially confined region. Additionally, electrostatic repulsion between the negatively charged DNA and the hydrogel matrix exerted a stretching force on ssDNA, further promoting its extended conformation. This disruption of the intramolecular secondary structures in the random coil form facilitated a more efficient hybridization, ultimately accelerating the reaction rate.

The specificity of the biosensor was assessed by analyzing the fluorescence response to various targets, including the correct *afl*D gene sequence, a single-base mismatch (MT1), a two-base mismatch (MT2), and a random ssDNA sequence. As illustrated in Figure 5C, the fluorescence intensities observed for MT1, MT2, and the random ssDNA sequence were comparable to those of the blank control and significantly lower than the response to the *afl*D gene. These findings confirmed that the proposed biosensor had high specificity for the *afl*D gene detection.

### Practicability of the Biosensor

From a practical perspective, it is crucial to assess the effectiveness of our biosensor for use in real-world samples. Therefore, the developed biosensor was employed to detect the *afl*D gene in corn flour obtained from a local market. The extraction of the *afl*D gene was carried out using a methanol–water mixture, followed by centrifugation and filtration through a 0.22 μm disposable syringe filter to remove particulates. The sample preparation steps were performed in accordance with a previously established protocol, as detailed in the Supporting Information.^58^

To assess the biosensor’s performance, we spiked the extracted samples with three different concentrations of the *afl*D gene (10, 5, and 0.05 μM) and utilized the developed biosensor for *afl*D gene detection using LSCM. As shown in Figure 5D, the biosensor successfully detected the target *afl*D gene in corn flour, indicating that the biosensor possessed excellent anti-interference properties and was well suited for early warning of AFB1 in food matrices. Additionally, a comparison was conducted with previously reported fluorescent biosensors for the *afl*D gene (Table 1), which revealed that our method achieved a detection speed over 160-fold faster than the fastest previously reported approach. Indeed, the LOD of our method is higher than the other amplification-based biosensors. However, those strategies often achieve lower LODs at the cost of multistep, labeled procedures. In contrast, our key contribution lies in its label-free nature and operational simplicity, enabling ultrafast detection with minimal reagents. This makes it uniquely suited for rapid, on-site applications where speed and ease of use are paramount. As the sensitivity of this method has room for improvement, future work will explore signal amplification strategies and probe optimization to enhance performance without compromising these core advantages.

**Table 1:** Comparison of the Proposed Biosensor with Previously Reported Methods for the Detection of the *afl*D Gene

strategy	LOD	linear range	detection time	ref.
FRET-based fluorescence biosensor using NCQDs and AuNPs	1.95 nM	10–150 nM	60 min	^59^
*Y*-shaped aptasensor for detection of the *afl*D gene	0.75 nM	0.5–500 nM	80 min	^25^
hairpin DNA-assisted FRET-based fluorescence probe switch for *afl*D gene detection	0.02 nM	0.05–200 nM	40 min	^24^
Ag^+^-mediated CHA-based fluorescence biosensor for *afl*D detection	1.66 pM	0.005–50 nM	60 min	^60^
impedimetric electrochemical DNA biosensor based on AuNPs	0.55 nM	1 nM–10 μM	4 h	^61^
space confinement-enhanced fluorescence detection of the *afl*D gene on a hydrogel particle array	19.05 nM	50–1000 nM	15 s	This work

## Conclusions

In this study, we developed a space-confined hydrogel particle-based biosensor for the rapid, label-free fluorescence detection of the *afl*D gene, enabling early warning of AFB1 contamination. By employing the space-confinement effect of PEG–COOH hydrogel particles, the system significantly enhanced the reaction kinetics by concentrating target DNA in the outer water layer, achieving ultrafast detection within 15 s, which was 240 times faster than conventional methods. The integration of GO could effectively minimize background interference, while the *V*-shaped dicationic fluorophore VLM ensured high specificity through selective binding to the dsDNA between the *afl*D gene and cDNA, accompanied by a strong fluorescence turn-on response. The biosensor demonstrated exceptional analytical performance with a LOD of 19.05 nM and a wide linear range of 50–1000 nM. Its rapidity, sensitivity, and specificity made it a powerful tool for on-site, real-time monitoring and early warning of AFB1 contamination in food supplies, addressing the limitations of traditional gene detection techniques that rely on complex instrumentation and time-consuming procedures. This work not only provided a practical solution for early AFB1 risk assessment but also opened new avenues for portable biosensing platforms in food safety, environmental monitoring, and point-of-care diagnostics. While these findings are highly promising, certain aspects remain to be further explored. The present work focused exclusively on the *afl*D gene, whereas extending the approach to additional aflatoxin biosynthetic genes may further strengthen its diagnostic scope. Moreover, although the interaction of VLM with dsDNA was systematically investigated, future studies could explore its potential application with higher-order DNA structures, such as triplexes and quadruplexes. Addressing these aspects would not only enhance the understanding of VLM-DNA recognition mechanisms but also provide valuable insights for designing more sensitive and selective biosensors applicable in complex biological and environmental contexts.

## Supplementary Material



## Notes

Materials; apparatus; preparation of spiked corn samples; determination of LOD; characterization of the hydrogel stability on the superhydrophobic substrate by rinsing with water; schematic of the distribution and arrangement of cDNA/VLM molecules on hydrogel particles in PEG–COOH hydrogels and in PEG hydrogels; and fluorescence images of 100 μg·mL^–1^ of GO droplets on fluorescent hydrogel particles after evaporation for 0 and 45 min at room temperature (PDF)
