# Supplementary material for: Space-Confined Hydrogel Particle-Based Biosensor for Early Warning of Aflatoxin B1 via Rapid Label-Free Fluorescence Detection of the aflD Gene
Source: Anal Chem. 2025 Nov 19;97(47):26024–33. doi: 10.1021/acs.analchem.5c04105 (PMC12676520; doi:10.1021/acs.analchem.5c04105)
Supplement: Supplementary file 1 [file ac5c04105_si_001.pdf]

Supporting Information

Space-Confined Hydrogel Particles-Based Biosensor for Early Warning of Aflatoxin B1 via Rapid Label-Free Fluorescence Detection of *afl*D Gene

Kawtar Ettayri, [†] Hailong Zhang, [‡] Wenwen Tian,[#] Lingliang Long,[†] Yu Chen,[†] Mengyao Ma,[†] Man Shing Wong, [*,‡] Kun Wang,[†] and Jing Qian [*, †]

[†] *School of Chemistry and Chemical Engineering, Jiangsu University, Zhenjiang 212013, PR China*

[‡] *Department of Chemistry and Institute of Advanced Materials, Hong Kong Baptist University, Kowloon Tong, Hong Kong*

[#] *Analysis and Testing Center, Southeast University, Nanjing 211189, P.R.China*

*Corresponding authors: E-mail addresses: mswong@hkbu.edu.hk (M.S. Wong); qianj@ujs.edu.cn (J. Qian)

List of contents

Materials.....S-3
Apparatus.....S-3
Preparation of Spiked Corn Samples.....S-3
Determination of LOD.....S-4
Scheme S1.....S-5
Figure S1.....S-6
Figure S2.....S-7
Figure S3.....S-8
Table S1.....S-9

Materials. 2-Amino-2-(hydroxymethyl)-1,3-propanediol (Tris) was purchased from Sigma. Polyethylene glycol diacrylate (PEG-DA, MW 170.16), 2-hydroxy-2-methyl-propiophenone (used as the photoinitiator), and acrylic polyethylene glycol carboxylate (Acryl-PEG-COOH, MW 3400) were sourced from commercial suppliers. Additional materials, including 1H, 1H, 2H, 2H-perfluorodecyltrichlorosilane (FDTS), polyethylene glycol (MW 200), graphene oxide, toluene, (3-aminopropyl) triethoxysilane (ATPES), and SiO_2, were obtained from Thermo Scientific (China). All solutions were prepared using ultrapure water (Milli-Q, 18.2 MΩ·cm). All DNA strands in this work were synthesized and purified by Shanghai Sangon Biotechnology Technology Services Co., Ltd. (Shanghai, China) and all sequence information has been shown in Table S1.

Apparatus. UV-vis absorbance spectra were recorded using a UV-2450 spectrophotometer (Shimadzu, Japan) at room temperature. Fluorescence spectra were acquired using an F-4500 fluorescence spectrophotometer (Tokyo, Japan). Fluorescence images were obtained on a Leica TCS SP5 II laser confocal scanning microscope (Leica, Germany). The morphology of the synthesized GO nanosheets was characterized using transmission electron microscopy (TEM) with a JEOL JSM-6700 instrument (Japan). The morphologies of the PEG hydrogel particles were characterized by scanning electron microscopy (SEM) using a JSM-7800F (JEOL Ltd., Japan). Water contact angles (CA) were measured on an OCA20 system (Data Physics, Germany) with a 5 μL water droplet (Milli-Q, 18.2 MΩ·cm) at ambient temperature. The surface treatment of substrates was carried out using a Plasma Cleaner PDC-MG (China). The distribution results of *afl*D gene were characterized by a laser scanning confocal microscope (LSCM, Leica TCS SP5).

Preparation of Spiked Corn Samples. Non-contaminated corn was obtained from the local supermarket, 11 g of the as-obtained corn flour were then mixed with 1 g of sodium chloride and then milled together. The corn flour was divided into three copies and spiked with *afl*D at distinctive concentrations. Subsequently, 10 mL of the extraction solvent (methanol: water=6:4 (v/v)) was introduced and the mixture was shake for half an hour. Being centrifugated at 6000 rpm for 10 min, the extract was then treated with a 0.22 μm syringe filter while pH value of the solution was tuned to be 7.4. The concentrations of *afl*D are 10, 5, and 0.05 μM.

Determination of LOD.

1- Mean of the blank $\overline{X}_{b1}$: The mean fluorescence intensity of the blank samples was calculated as follows:

$$\overline{X}_{b1}=\frac{10.6985+9.3015+10}{3}=10$$

2- Standard Deviation S.D. The standard deviation of the blank fluorescence intensity was computed using:

$$\text{S.D}=\sqrt{\frac{1}{n\text{-}1}\sum_{k=1}^{n}\left(X_{\text{blank}}\text{-}\overline{X}_{b1}\right)^2}=\sqrt{\frac{[(10.6985\text{-}10)^2+(9.3015\text{-}10)^2+(10\text{-}10)^2]}{2}}=0.6985 \text{ units}$$

According to the analytical results in Figure 4C, the linear regression equation of the fluorescence aptasensor can be expressed as:

$$I = 39.68 + 0.11C_{afl\text{D/nM}}.$$

Where I is the fluorescence intensity, and c is the concentration of the target, the LOD could be expressed as follows:

3- Determination of LOD:

$$\text{LOD} = \frac{3 \times S.D}{K} = \frac{3\times0.6985}{0.11} = 19.05 \text{ (nM)}$$

where k is the slope of the curve equation equal to 0.11, and S.D. represents the standard deviation for the fluorescence intensity of VLM in the absence of *afl*D gene.

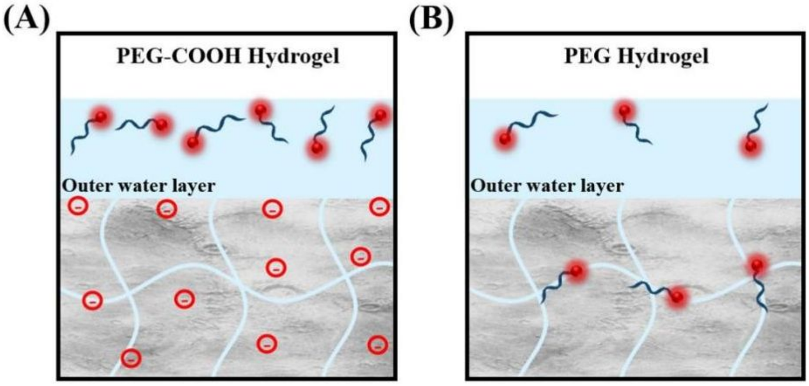

Scheme S1. Schematic of the arrangement of the VLM/cDNA complex on hydrogel particles and the effect of charges on its distribution in (A) PEG-COOH and (B) PEG hydrogels.

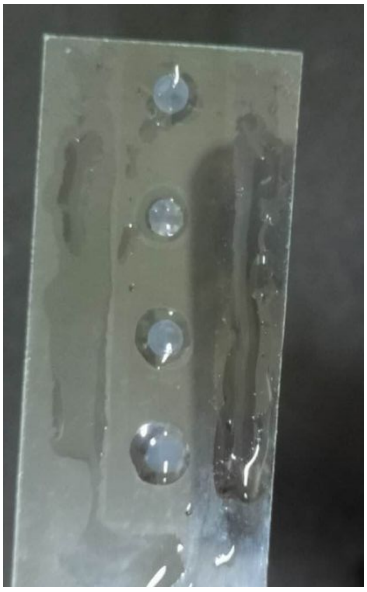

Figure S1. Characterization of the hydrogel stability on the superhydrophobic substrate by rinsing with water. After being rinsed with water, the hydrogel particles can still be stably anchored on the substrate.

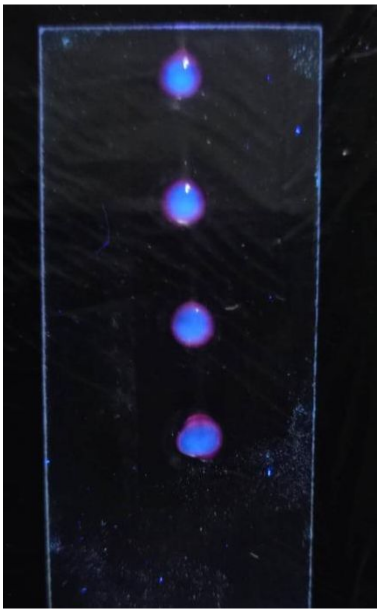

Figure S2. The Microarray of GO-cDNA/VLM/*afl*D gene complex in superwettable micropattern.

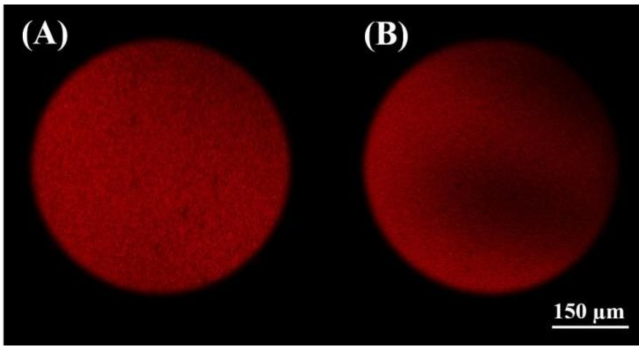

Figure S3. Fluorescence images of 100 µg mL^{-1} of GO droplets on fluorescent hydrogel particles after evaporation for 0 min (A) and 45 min (B) at room temperature.

Table S1. The Oligonucleotides' Sequences for Use in This Work.

DNA Type	Sequence (from 5′ to 3′)
*afl*D gene	GGA TCT CAA CTC CCC TGG TAG
cDNA for *afl*D gene	CCT AGA GTT GAG GGG ACC ATC
MT 1	GTA TCT GAA CTG CCA TGG TAG
MT 2	GGA TGT CAA CTC CGC TGG TAG
Random ssDNA	CTG CAT GTA GTA ATG CCA CGT
